# Promoting Safety through Well-Being: An Experience in Healthcare

**DOI:** 10.3389/fpsyg.2016.01208

**Published:** 2016-08-12

**Authors:** Andreina Bruno, Fabrizio Bracco

**Affiliations:** Department of Educational Sciences, University of GenoaGenoa, Italy

**Keywords:** healthcare, safety, well-being, human error, reporting, surgery, participatory action-research

## Abstract

Practitioners’ well-being and clinical risk management are two interrelated concepts in healthcare. Patient safety, workers’ safety and practitioners well-being have often been managed and measured with different methods, even though they are tightly linked. In this paper we propose a method that is suitable to increase organizational health. The action-research project aims to increase the commitment of healthcare managers and practitioners toward the development of an organizational culture which is oriented to patient and practitioner safety and well-being. These are crucial organizational resource for an effective process management. The project lasted 18 months and involved 60 nurses and physicians working in the operating room of six hospitals in the North of Italy. The project aimed to develop an inter-organizational methodology for noticing and monitoring critical threats to safety and well-being. The tool consisted of a report form in which practitioners could describe possible threats, solutions and personal contributions to the solutions. The participants designed it according to their practice and it was considered suitable and usable in their current work activities. Its added value is to overcome the habitual bottleneck between anomalies investigation and action planning, by identifying a specific role in the learning process to take care of the transition from data gathering to data use. The tool aims to enable individuals and teams to monitor and share ideas about critical aspects that affect their safety and well-being, collect contributions to solve them, sustain dissemination of good practices and frame health promotion as a crucial organizational resource.

## Introduction

Since the publication of the famous report “To Err is Human” (Kohn et al., 2000), the issue of medical error has gained increasing attention and has been widely investigated. The report demonstrated the incidence of human error as a main root cause of patients injuries and deaths, recording that every year, in the United States, between 44.000 to 98.000 people lose their lives because of factors concerning preventable errors in clinical treatment. All western countries share the same statistics, thus leading researchers in clinical risk management to argue that the problem resides in how complex organizations deal with likewise complex issues as those concerning healthcare (Hollnagel et al., 2013). The category of “human error” is extremely vague and misleading, since it can comprise almost everything (Dekker, 2007). A closer look inside this category reveals that doctors and nurses do not make mistakes because they are unprepared, unprofessional, or they do not care about patient’s health. Rather, they make mistakes because they often lack proper coordination, communication, and leadership in diagnosis and treatment (Leape et al., 1993; Hollnagel, 2014). Recent evidence demonstrates the link between workers’ well-being and patient safety, since burnout, workload, miscommunication, and dysfunctional organizational climate could be the factors leading to poor performance in complex environments like healthcare (Yassy and Hancock, 2005; Henriksen and Dayton, 2006; Hoffman and Mark, 2006; Laschinger et al., 2006; Elfering et al., 2007; Reader et al., 2008). According to the Joint Commission (2016) report about sentinel event data, the top-three root causes for patients’ harm, between 2013 and 2015, are human factors, leadership, and communication. These factors have been recently framed as non-technical skills (Flinn et al., 2008), since they do not strictly concern the technical expertise but, rather, they are based on social and cognitive abilities that help the team to cooperate and perform safely and effectively (Gordon et al., 2012). In addition, a close relationship between non-technical skills and performance has been demonstrated (Bower et al., 2003; West, 2004), since stress, organizational malaise, miscommunication, and workers’ isolation are connected to dysfunctional resource management, poor coordination and underperformance (Arenas et al., 2015). This relation can be framed according to the well-known pyramid of accidents, where for each fatality (the tip of the pyramid) there could be hundreds of near-misses and thousands of at risk behaviors (the larger base; Heinrich, 1931). Within an organization, the sharp-end workers (practitioners at the front-line) are the only one able to notice and report information pertaining the lower part of the pyramid, while managers will be aware only of the tip of the pyramid, when the injury or the loss is impossible to hide (Wreathall, 2006). Therefore, safety culture is possible only when there is a clear commitment toward workers’ well-being, since they will have the resources and motivation to report data in the lower part of the pyramid (i.e., the weak signals concerning risky situations and near misses).

The most widely used method for sharing information about errors and threats is incident reporting. It is based on a free and often anonymous description of what happened and the event’s supposed contributing factors. The document is then analyzed by risk managers in order to act on the factors that led to the unwanted outcome. Notwithstanding its potential for safety, the rate of reporting is often lower than expected (Whitaker and Ibrahim, 2016). The reasons could be the need for anonymity, the lack of feedback, a poor reporting culture, the complexity of procedures, the confusion about what and how to report, the lack of organizational commitment toward reporting (Vincent et al., 1999). A similar underuse is reported among Italian healthcare workers, and the reasons concern the blame attitude in analyzing errors, fear of mistrust from colleagues, the complexity and overlap of reporting tools, and skepticism toward the benefit of the tool (Albolino et al., 2010). According to Weick and Sutcliffe (2007), incident reporting is not effective for safety if it is not supported in advance by other tools that focus on weak signals and treat anomalies and human error as a resource for organizational learning and not something to hide and blame. In addition, if the goal is to enhance a proper and widespread use of the tools, we claim that they need to be developed through a participatory process embedded in a specific context (Bracco et al., 2011). This is confirmed by the most recent evidence that shows that lean management increases only productivity without positive impacts on healthcare outcomes (Andersen et al., 2014).

The aim of the paper is to propose a method that is suitable to increase the learning culture in organizations where the reporting culture is weak. We present an action-research project that aims to sustain healthcare workers and managers in the development of an organizational culture oriented toward safety and well-being. Safety and well-being were framed as: (i) the main ingredient to maintain the quality of services and the quality of organizational life in the hospital, both for practitioners and for patients, and (ii) supported by tools that focus on weak signals and treat anomalies and human error as a resource for organizational learning.

The proposal was to widen the learning zone on professional practice to introduce cultural changes: it means to move the organizational sensitivity from the top to the base of the accident pyramid.

The specific goals of this project were:

(i) To frame safety as an outcome of organizational well-being;
(ii) To develop practitioners’ reflective practices and the dissemination of good practices among the colleagues;
(iii) To sustain the participatory development of a tool for the detection of potential threats, their analysis and monitoring.

## Method

### Context and Participants

The context is the health care domain, specifically surgery. The project involved 60 nurses and physicians, working in the operating room of six hospitals in the North of Italy. The project was deployed at two different levels:

(i) Project group involved 25 practitioners that worked at the design stage and the implementation stage, through plenary and team meetings, where they engaged their colleagues by using the tool designed during the project;
(ii) Large group involved the above mentioned group and a further 35 doctors and nurses who belonged to the same hospitals, together with hospital risk managers. This second level participated in the initial phase of the project, in the presentation of the project outcomes, and in the last public workshop.

The project lasted 18 months, from June 2010 to February 2011.

### Phases of the Project

The whole project was based both on reflection on practices (Bruno et al., 2011) and on innovative design and on the field experimentation.

The phases are:

(i) Focus group on health, well-being, safety, and professional practice;
(ii) Presentation of the results of the focus group and discussion on the cultural perspectives on safety and well-being;
(iii) Analysis of work practices through the critical incident technique (Flanagan, 1954);
(iv) Planning of safety and well-being strategies aimed at the development of a tool for the monitoring of practices (as will be described later, this tool was a risk reporting system);
(v) Tool’s usability assessment;
(vi) Evaluation of the project’s outcomes and participants’ experiences;
(vii) Public workshop for the dissemination of the project’s outcomes.

### Procedures

Several documents were written during the project: meetings memorandum (52-page document), project teams materials, focus group report. A thematic analysis of these documents was conducted.

## Outcomes

The analysis reveals three main thematic areas:

(i) Well-being and safety culture,
(ii) The participatory process,
(iii) The use of the tool.

### Well-being and Safety Culture

Findings reveals that safety and well-being are considered by participants as two intertwined concepts:

“… the definition of well-being is part of and completes the definition of safety,”

“…working under well-being conditions enables people to work safely…,”

“… the definitions of the safety and well-being are based on a single answer….”

And that safety is meant as related both to the patient and the operator:

“… protecting ourselves and the patients at the same time…,”

“…be aware of the actions outcomes, both on me and on the others….”

Training and reflection on practices are considered relevant for dampening risks:

“… we need to invest in training in order to provide a high performance standard…,”

*“… knowing what I am doing and knowing the tools I am using, being aware of my actions and their consequences both on me and the others…,”* because sometimes *“we keep our fingers crossed… and we hope that no emergency will happen….”*

The reporting practice is considered useful:

“… it helps in understanding factors that led to the mistake…,”

“… the reporting helped us in accomplishing some results….”

However, the reporting culture is quite weak because:

“… we do not have the culture to freely speak about errors…”

and incident reporting tools are mainly used for serious events. Practitioners do not perceive the immediate benefit of the reporting, since the feedback is often absent, partial or late. In addition, they believe that safety is a matter of culture and it needs a systemic change that should not burden just front-line operators.

They claim their need to manage “small” everyday problems in order to pay more attention to error management:

“… solving small problems makes things easier… it’s like a trickle eroding a mountain, if you don’t stop that trickle, sooner or later the mountain will disappear….”

The everyday problems are concerning relationships, communication, and organization issues. They are considered the most critical factors because when they are chronic the operators’ well-being is perceived at risk.

### The Participatory Process

Participants stressed the need to increase organizational well-being and, together with this, the quality of the service provided. These were the premises for the development of a participatory process that aimed to design a tool for the detection and monitoring of critical situations for safety and well-being. This tool (named Critical Situations Detection and Monitoring Form) allowed the participants to clarify problems and their origins and the potential consequences in terms of well-being and safety. Moreover, they were able to clarify possible solutions and identify who could be in charge to implement them. This tool aimed to report critical conditions that act against well-being, since practitioners clearly stated that safety-oriented behaviors could be accomplished in conditions of well-being.

The tool design went through several steps (**Table 1**). The preliminary step aimed to probe the acceptability of a tool to monitor well-being. This first document was developed according to the incident reporting form for risk management in the operating room. After that, the group revised the checklist in order to simplify it, allowing users to have a quicker and seamless access to questions in the form. Only in a further phase the participants moved their attention from the incident (the tip of the pyramid) to the weak signals (the base of the pyramid). The participants claimed that an effective risk management (both for patients and practitioners) is an essential condition for well-being, therefore they designed the tool with the goal of reporting also potential threats and near-misses. This tool does not aim to substitute other reporting tools already used in case of adverse events, actually, it should be used together with them, because it has a broader perspective. Practitioners’ safety and well-being were therefore tackled with a tool divided in two sections: (i) Critical situation detection form, and (ii) Solution analysis form. The two sections of the tool are therefore part of a systemic intervention that aims to promote safety and well-being. The first section, where the critical situations are reported, allows operators to share information about their needs, provides them with the responsibility of reporting anomalies and critical conditions in their workplace. Front-line practitioners are the only ones in direct contact with everyday practices, they know the methods used to implement procedures and the critical factors that, over time, can erode safety and well-being. The second section is aimed at making the analysis of the problems reported in the first section explicit. It allows practitioners to find a solution (whenever possible) that is as quick as possible, suitable for their contexts, oriented by internal needs and not controlled by external factors.

**Table 1:** The steps of the participatory design.

Steps	Outcomes
1. Incident reporting form	Adaptation of a pre-existing form for risk management in the operating room
2. Criticalities reporting form	Form for the reporting of potential threats and near misses
3. Splitting of the form	A tool divided in two sections: (i) Critical situation detection form, (ii) Solution analysis form.
4. Addition of a third section	A tool divided in three sections: (i) Critical situation detection form, (ii) Solution analysis form, (iii) Monitoring of the solution process.
5. Electronic version of the tool	Spreadsheet with three sections: (i) Anomalies detection, (ii) Problem setting and problem solving, (iii) Solution process monitoring.
6. Tool evaluation	Evaluation based on AGREE (2003) criteria for the assessment of the quality of clinical guidelines: (i) clarity of the overall objective, (ii) the target population, (iii) the stakeholder involvement, (iv) tool applicability, (v) resource implications.

Only after the development of the two sections and a preliminary testing, participants proposed to add a third section to the tool. This is about the monitoring of the process which aims to solve the critical situations that were reported and analyzed in the previous sections. In addition, this section can highlight the weak points of the network that is engaged in solving the problems, because it analytically describes the steps of the process and those people in charge of problem management. Sometimes problems are complex and need an iterative process of adjustment of the solution. It is often necessary to reuse these modules several times until there is sufficient agreement and satisfaction about the problem solution.

This three-folded tool could now provide the relevant information about each step of the process and demonstrates that “learning from critical situations” would fail if the process stopped at the first phase, while all three steps are crucial to accomplish effective results. Sections 2 and 3 are therefore what make this tool something different to a traditional incident reporting form, where practitioners are asked to provide data but have no awareness on the problem analysis and any role in its solution.

At the end of the project, participants decided to implement an electronic version of the tool, adapting it to a spreadsheet form. It was designed as a 3-module tool and each section corresponded to one of the 3 phases of the process: (i) Anomalies detection, (ii) Problem setting and problem solving, (iii) Solution process monitoring.

Eventually, participants proposed to evaluate the tool according to the AGREE criteria for the assessment of the quality of clinical guidelines (AGREE, 2003): clarity of the overall objective, the target population, the stakeholder involvement, the evaluation of applicability and resource implications, etc. According to this framework, participants were able to differentiate this tool from already existing methods, tools and practices, and to appreciate its synergy with them.

### The Use of the Tool

Participants involved their colleagues, coordinators, administrators, and finally professional associations in the project, in order to develop the tool, and then make the intermediate results visible.

The project teams related several qualitatively rich experiences in using, monitoring and disseminating the reporting practice; indeed, their overall satisfaction highlighted the specificity of the tool compared to other methods.

In some contexts, the operators expressed strong desire to implement the project, revealing their need to share their experiences about the hardship of daily professional practice. In these cases, the participatory model allowed them to shift from a “complaint” register to an “activation” one. In particular, the involvement of the team coordinator and other institutional key-roles (such as Risk Manager, Quality Manager, Occupational Health Physician, Occupational Health and Safety Manager, Nursing Coordinator) has been a crucial point.

However, where the tool’s use presented difficulties, the following critical issues emerged: differences in vision and approach to well-being, poor management clarity in relation to the mandate, and concerns about data misuse. We have also been informed that, in a hospital, the management did not authorize the fieldwork phase. In addition, during the first meeting, some operators expressed their absolute disbelief in the possibility of using data in a non-blaming way and abandoned the project.

## Discussion

The participatory model seems to be suitable to sustain the participants’ engagement and responsibility of well-being and safety management. In particular, by starting from the analysis of practices and not from a predefined model, the project allowed the participants to enable open confrontation about inter-professional practices (nurses, anesthetists, surgeons), confrontation among practices in different contexts (health units, hospitals) and among different roles (coordinators, safety managers, etc.), and dissemination of good practices. The development of the operators’ competencies in reflecting upon their practices and sustaining a participatory process of organizational solutions enables them to reduce their learned helplessness and resignation and to design a report form according to their practices, which was considered suitable and usable in their current work activities.

This participatory model seems to counterbalance incident reporting limits and poor reporting culture (Whitaker and Ibrahim, 2016). In fact, this tool has several advantages: it involves operators in a process of information flow; it reduces the complexity of the reporting practice, which is developed by the user itself; it enables them to manage the process. Feedback is no longer delegated to others, since the reporting system is a traceable process that unifies the problem diagnosis phase and the solution implementation assessment. From this perspective, it is possible to overcome the common bottleneck between anomaly investigation and action planning (Drupsteen et al., 2013), by identifying a specific role in the learning process to take care of the transition from data gathering to data use. Accordingly, the participatory model gives operators the perception of responsibility and ability to intervene and modify their environment. In addition, the tool becomes a database of already solved issues concerning safety and well-being, allowing them to learn from past experiences.

Furthermore, in this kind of participatory process, there are two instances to take into account. Not only is the bottom-up process development important, the same attention has to be devoted to the top-down commitment for the tool implementation. If correctly balanced, from the proximity to the front-end practices, the advantage is the adherence to the current work activities and the contextualization of the tool within actual practices and regulations.

Managers’ commitment for tool implementation is necessary to provide quick and credible feedback, when required, and, most of all, to acknowledge and promote new practices into the organizational culture. Indeed, in contexts where the monitoring process that unifies the problem diagnosis phase and the solution implementation assessment failed, the tool’s implementation was not institutionally supported, notwithstanding the initial commitment of the top management. Such a need to sustain the long-term project is evident in the participants’ involvement of their colleagues and coordinators. The aim of such efforts is to continuously sustain the good practices realized by the operators, i.e., organizational criticality management for the improvement of safety and well-being of both workers and patients.

## Author Contributions

All authors listed, have made substantial, direct and intellectual contribution to the work, and approved it for publication.

## Conflict of Interest Statement

The authors declare that the research was conducted in the absence of any commercial or financial relationships that could be construed as a potential conflict of interest. The reviewer JP and the handling Editor declared their shared affiliation, and the handling Editor states that the process nevertheless met the standards of a fair and objective review.
